# Accessing good health information and resources

**Published:** 2017-05-12

**Authors:** Sally Parsley

**Affiliations:** 1 E-communications manager, International Centre for Eye Health, London School of Hygiene and Tropical Medicine, London, UK.

**Health workers need to be able to access health information and resources to update and apply their knowledge and skills and continue their professional development.**

**Figure:**
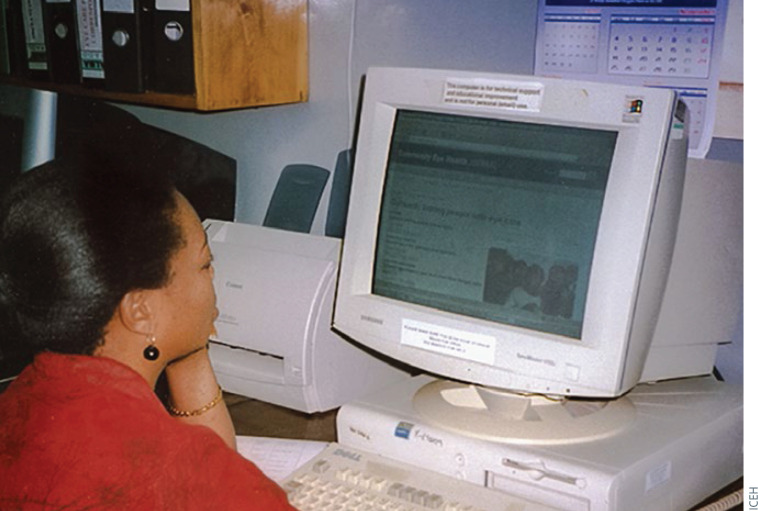
Learning online. AFRICA

Making health information available and usable to all is a complex process not yet adequately addressed (see Figure 1). It has to be appropriate, high quality, timely, easy to understand, relevant for the location it will be used in, and provided in an appropriate format. For example, you cannot learn a new surgical skill by reading about it, a much better method is to take a course or watch a video, preferably one suited for the local need.

Where do you find these resources and opportunities? Information, communications technologies (ICTs) such as the internet are a promising mechanism to help address the health workforce information needs. Health workers need access to ICTs but they also need strong information and computer skills to search, select and make use of the available information and resources.^1^

Availability of high-quality, up-to-date and locally relevant materials is limited in many settings and there is a lack of investment and organisational support for developing information and computer skills and the infrastructure needed to access printed and digital information.

In every setting, major health stakeholders need to continue to develop and implement knowledge management strategies to enable health workers to use the evidence-based information and knowledge available to them.^2^,^3^

The following infographic aims to guide eye health clinicians, educators, managers and leaders on:

- Identifying the information need
- Developing a search strategy
- Carrying out an effective online search
- Finding sources of good eye health information and resources on the internet

**Figure 1:**
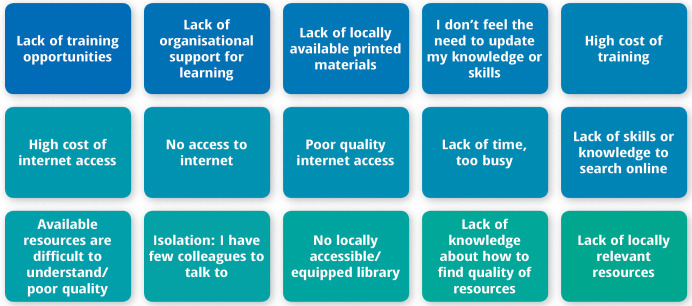
Which health information challenges do you face? How do you overcome them?

## A Identifying the information need

**Figure:**
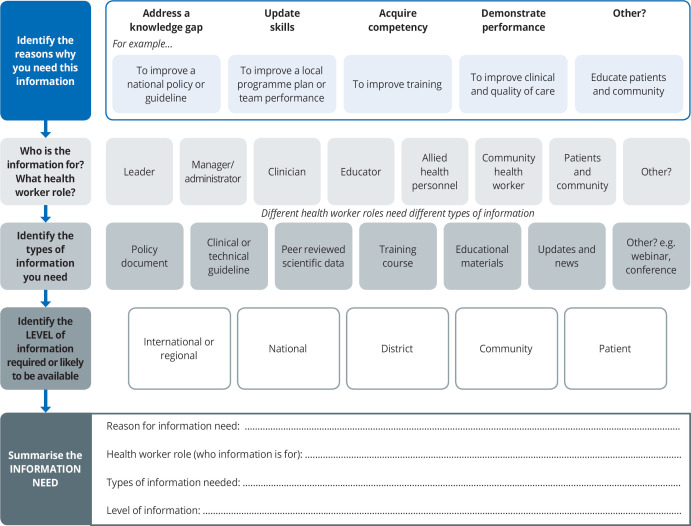


## B Developing a search strategy

**Figure:**
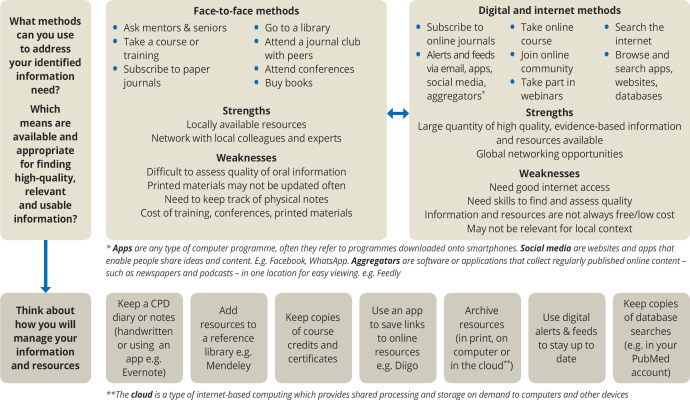


## C Carrying out an effective online search

1. Extract the **keywords and phrases** from your identified information need (see section A).
2. Identify which **search engine** to use. Internet search engines (such as Google) will return wide results but with variable quality.
3. Enter your keywords and phrases into the engine.
4. **Select and evaluate results which seem relevant.** Review the summary or abstract and exclude irrelevant or low quality resources. Ask yourself:
  - Who published this resource? Does the publisher have a good reputation? Has it been peer-reviewed for quality?
  - When was it published? Is it up to date?
  - Is the information suitable for use in your setting?
  - Is the resource ‘Open’? Can it be downloaded and shared for free? Or do you need to pay?
  - Is the technical production good? Can you, or anybody, access and use it easily?
5. **Review the relevant resources** in detail. E.g. read the whole article. If necessary, make notes of the most relevant information from each source. For complex information needs, integrate your notes into a matrix to help you track your ideas and relate back to your topic.
6. **Manage your notes and information** you have found (see section B).

## D Good sources of free and low cost eye care information and resources on the internet

*What have we missed out? Send suggestions to **editor@cehjournal.org** or to CEHJ Twitter or Facebook and we will review and share them in later issues*.

**National and local sources**

Eye care bodies in your country may provide useful health information and CPD opportunities. For example:

- Bhutan Medical and Health Council **www.bmhc.gov.bt**
- Ophthalmological Society of Nigeria **https://osnig.org**
- India national programme for control of blindness **http://npcb.nic.in**

There may be professional interest groups you can join – face-to-face or by email or social media e.g. Facebook or WhatsApp

### Global data, policy and guidelines

- World Health Organization Prevention of blindness **www.who.int/blindness/en**
  Key international policies, data and guidelines
- IAPB Vision Atlas **http://atlas.iapb.org**
  Country level maps and data on avoidable blindness and sight loss.
  From the International Agency for the Prevention of Blindness
- Trachoma Atlas **www.trachomaatlas.org**
  Online global atlas of the distribution and prevalence of trachoma.

### Free online courses

- International Centre for Eye Health courses **http://iceh.lshtm.ac.uk/oer**
  Public health courses on Global Blindness: Planning and Managing Eye Care Services, Ophthalmic Epidemiology, Eliminating Trachoma and Diabetic Retinopathy (coming soon)
- Cybersight courses **https://cybersight.org/online-learning**
  A number of introductory clinical courses. Provided by ORBIS
- Aurosiksha **www.aurosiksha.org**
  Short courses on eye care management from Aravind Eye Care System

### Scientific databases

- Medline/PubMed **www.pubmed.gov**
  PubMedCentral **www.ncbi.nlm.nih.gov/pmc**
  An index of the world's biomedical literature from the National Library of Medicine, USA. PubMedCentral indexes Open Access literature
- Cochrane Eyes and Vision Reviews **http://eyes.cochrane.org/links-our-reviews** – systematic reviews of the current scientific evidence on interventions to treat or prevent eye diseases or visual impairment.

### Regional journals with free access

- Indian Journal of Ophthalmology **www.ijo.in**
- Journal of Ophthalmology of Eastern, Central and Southern Africa **www.coecsa.org/ojs-2.4.2/index.php/JOECSA/index**
- Middle East African Journal of Ophthalmology **www.meajo.org**

### International and regional training and CPD providers

- Aravind Eye Care System Education and Training **www.aurovikas.co.in** Clinical and non-clinical training for all levels
- College of Ophthalmology of Eastern Central and Southern Africa **www.coecsa.org**
- International Council of Ophthalmology (ICO) **www.icoph.org** Foundation, standard and advanced level exams
- Joint Commission on Allied Health Personnel in Ophthalmology: Global Center for Online Ophthalmic Continuing Education **http://eyecarece.jcahpo.org**
- Training institutions for eye health professionals in Africa. The IAPB Africa database. **http://www.iapbafrica.co.za/resource/resourceitem/808/1**

### Educational materials: Libraries and databases

- Cybersight Library **cybersight.org/portfolio**
  Clinical quizzes, video lectures and textbooks
- Eye Rounds **eyerounds.org**
  Case reports, photographs, tutorials. From the University of Iowa.
- IAPB Africa Resources **www.iapbafrica.co.za/resource/index/1**
- ICO resources **www.icoph.org/resources.html**
  ICO resources for educators **http://educators.icoph.org**
  Hundreds of useful links for ophthalmologists and educators
- VISION2020 e-resource **v2020eresource.org**
  Resources on eye care management. From Aravind Eye Care System.

### Image and video repositories

Eyerounds Atlas **www.eyerounds.org**

Community Eye Health Flickr Photostream **www.flickr.com/photos/communityeyehealth**

### Eye care apps

There are a number of free and low cost apps in ophthalmic education. Search for them on your app store. (See 2015 article from the AAO for ideas: “Top Ophthalmology Resident Apps” **http://bit.ly/2flciBV**)

**The HINARI – Access to Research Initiative** provides not-for-profit institutions in low- and middle-income countries with free or very low cost access to biomedical and social science journals. **www.who.int/hinari/en/**

**Massive Open Online Courses (MOOCs)** are free to take with some optional fees e.g. for accreditation. MOOCs bring hundreds or even thousands of people together to learn about a subject. 6850 MOOCs were available by the end of 2016 from providers such as Coursera and EdX (USA), FutureLearn (UK), XuetangX (China), Miríada X (Ibero-Americas), Edraak (Arabic) and Swayam (India). The Global Blindness course (see page 10) is run as a FutureLearn MOOC once or twice a year. Register your interest at **www.futurelearn.com/courses/global-blindness**

Class Central currently maintains one of the most up-to-date lists of MOOCs. **www.class-central.com**

**Open Educational Resources (OERs)** are learning materials free to anyone to access, reuse, adapt and share with others without having to seek permission from the original publisher. OERs are also called OpenCourseWare. A number of regional and health related OER repositories have been published: For example: OER Africa **www.oerafrica.org** or MIT and John Hopkins Public Health OpenCourseWare sites – **ocw.mit.edu/index.htm** and **ocw.jhsph.edu**
